# Ionic Liquid-Nanostructured Poly(Methyl Methacrylate)

**DOI:** 10.3390/nano9101376

**Published:** 2019-09-26

**Authors:** Clarice Fedosse Zornio, Sébastien Livi, Jannick Duchet-Rumeau, Jean-François Gerard

**Affiliations:** Ingénierie des Matériaux Polymères, Université de Lyon, CNRS, UMR 5223, INSA Lyon, F-69621 Villeurbanne, France

**Keywords:** ionic liquids, PMMA, plasticizer, mechanical behavior, crazing, thermal behavior

## Abstract

Here, ionic liquids (ILs) based on imidazolium and ammonium cations were used as modifying agents for poly(methyl methacrylate) (PMMA) by extrusion. The effects of the chemical nature of the cation and/or counter anion on the resulting properties of IL-modified PMMA blends were analyzed. It was found that the use of low amounts of ILs (2 wt.%) improved the thermal stability. A plasticizing effect of ILs is evidenced by a decrease in glass transition temperature Tg of the modified PMMA, allowing to get large strains at break (i.e., up to 280% or 400%) compared to neat PMMA. The deformation and fracture mechanisms of PMMA under uniaxial tensile stress (i.e., crazing) reveal that the presence of IL delayed the strain during the initiation step of crazing.

## 1. Introduction

In the field of engineering polymer materials, poly(methyl methacrylate) (PMMA) is a polymer material that is considered in many applications because of its low density, high optical clarity, high rigidity, easiness for processing, as well as its excellent chemical and ultraviolet resistance [1,2]. Thus, PMMA has been integrated in numerous applications such as medical devices, glass replacement, automotive parts, and so on [3,4,5,6,7,8,9]. However, PMMA is a brittle polymer material that has a low fracture toughness, which limits its uses [10]. In order to enhance its properties, different routes have been investigated: (i) the incorporation of inorganic particles such as silica, alumina, layered silicates, calcium carbonate, or carbon nanotubes [11,12,13,14]; (ii) the use of a dispersed rubber phase [15,16,17] or the blending with other thermoplastic polymers such as polyethylene (PE) and polycarbonate (PC) [18]; and (iii) the introduction of low molar mass components that act as plasticizers [19,20]. The addition of silica nanoparticles is a very promising route to enhance the thermal and mechanical properties of PMMA [21,22]. Different authors highlighted that the incorporation of silica nanoparticles could lead to a significant improvement of fracture toughness of the PMMA matrix [23,24].

Recently, ionic liquids (ILs) have appeared as new additives in polymers, allowing the design of advanced polymer-based materials [25]. ILs are organic salts that exhibit a low melting temperature, good ionic conductivity, good thermal stability, and negligible vapor pressure [26,27,28]. In fact, ILs were commonly used as suitable compounds for compatible agents of polymer blends [29,30,31,32], such as (nano)structuration agents of fluorinated polymers and copolymers [33,34], non-conventional initiators of epoxy reactive systems [35,36], as well as surfactants or plasticizers of different polymers [37,38,39,40]. Recent works reported the advantages ILs used as plasticizers in synthetic and biosourced polymers. The ability to act as plasticizers for imidazolium-based ILs was also reported for starch. In fact, 1-butyl-3-methylimidazolium chloride ((C_4_mIm)(Cl)) IL contributes in screening the numerous hydrogen bonds leading to a significant decrease of the glass transition temperature, Tg, and acts as a processing aid in the molten state of thermoplastic starch [38]. Others authors have investigated the influence of trihexyl(tetradecyl)phosphonium bis(trifluoromethylsulfonylimide) ((P_6,6,6,14_)(TFSI)) and 1-pentyl-3-methylimidazolium hexafluorophosphate ((C_5_mIm)(PF_6_)) ILs in poly(vinyl chloride) (PVC) [41] and poly(lactic acid) (PLA) [42], respectively. Rahman et al. also demonstrated that ILs based on ammonium, imidazolium, and phosphonium cations could be an alternative to traditional plasticizers of PVC, as such compounds have a diffusion rate (i.e., better leaching and migration resistance) [37]. Concerning amorphous polymers being glassy at room temperature, Scott et al. demonstrated the ability of imidazolium-based ILs to induce plastic deformation in PMMA. In fact, one of the conventional plasticizer agents used in PMMA (i.e., dioctyl phthalate, DOP) has a molecular structure close to the imidazolium cation, (i.e., an aromatic ring with saturated alkyl chains) [43,44].

As a consequence, the main goal of this work is to study the effect of a small quantity (2 wt.%) of ILs on the physical properties of IL-modified PMMA. Moreover, the influence of the chemical nature of the cation and/or anion on the morphology, thermal stability, and surface properties as well as the mechanical properties of PMMA have been studied. Deeper attention was brought for the first time, to our knowledge, to the crazing mechanism in PMMA under uniaxial tensile stress in order to identify the basic mechanisms involved in the large strain mechanical properties of IL-modified PMMA.

## 2. Materials and Methods 

### 2.1. Materials and Characterization Methods

**Figure 1:**
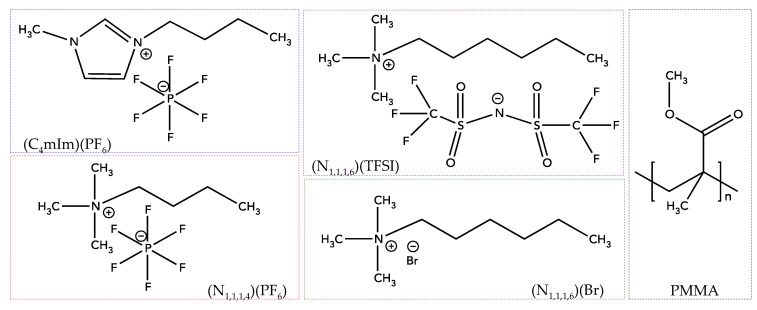
Chemical structures of ionic liquids (ILs) considered in this study. PMMA, poly(methyl methacrylate); TFSI, bis(trifluoromethanesulfonyl)imide; PF_6_, hexafluorophosphate; Br, Bromide.

PMMA was provided by Oroglas (Arkema Group). The molar masses were determined by Size-exclusion chromatography (SEC) with values of 30,300 and 36,000 g·mol^−1^, respectively. Imidazolium and ammonium-based ILs were supplied by Solvionic Co. and were denoted as 1-butyl-3-methylimidazolium hexafluorophosphate ((C_4_mIm)(PF_6_)), N-trimethyl-N-butylammonium hexafluorophosphate ((N_1,1,1,4_)(PF_6_)), N-trimethyl-N-hexylammonium bromide ((N_1,1,1,6_)(Br)), and N-trimethyl-N-hexylammonium bis(trifluoromethanesulfonyl)imide ((N_1,1,1,6_)(TFSI)). All the structures of ILs and PMMA are given in Figure 1. In addition, the designation of ILs as well as their melting temperatures, T_m_, and molar masses, M_m_, are reported in Table 1.

Fourier transform infrared spectroscopy (FTIR) spectra were recorded at 25 °C using a Nicolet iSO10 ThermoScientific spectrometer equipped with an Attenuated Total Reflectance (ATR) mode setup from 4000 to 600 cm^−1^.

Differential scanning calorimetry (DSC) analyses were performed using a Q20 TA instrument (TA Co. Ltd., New Castle, DE, USA). The samples were investigated from 193 to 453 K at a heating rate of 10 K·min^−1^ under N_2_ flow (50 mL·min^−1^). Before analysis, the samples were heated to 393 K to erase the thermal history. From DSC measurements, the glass transition temperature, Tg, was determined.

Thermogravimetric analysis (TGA) was performed using a Q500 thermogravimetric analyzer from TA Instruments. All samples were heated from 298 to 1173 K at different heating rates under inert atmosphere. T_onset_, T_max_, and T_final_ temperatures were determined. The plots ln(β/T2) versus 1/T obtained from TGA traces for each mass degradation and heating rate provided the activation energy of the degradation mechanism [45]. The degradation kinetics were analyzed according to the Kissinger–Akahira–Sunose method (KAS) equation [46].

$$\ln\left(\frac{\beta }{T^{2}}\right)=\mathrm{Constant}\;-\;\frac{E_{a}}{\mathrm{RT}},$$

where β is the heating rate (in K·min^−1^), T is the temperature (in K) recorded at each degree of degradation (defined as the fraction of the total mass loss in the process, ranging from 10 to 90%), R is the universal gas constant, and *E*_a_ is the activation energy (in kJ·mol^−1^).

Transmission electron microscopy (TEM) was performed at the Technical Center of Microstructures, at University of Lyon, using a Phillips CM 120 field emission scanning electron microscope (Philips, Amsterdam, The Netherlands) with an accelerating voltage of 80 kV. First, the samples were sliced using an ultramicrotome (Leica, Weitzlar, Germany) equipped with a diamond knife to obtain ultrathin sections 60 nm thick. Then, they were set on cooper grids for observation.

The surface energy of PMMA/IL materials was determined from the sessile drop method using a DataPhysics Instruments (GmbH) OCA 20 (Filderstadt, Germany). Water and diiodomethane were used as probe liquids for contact angle measurements. The nondispersive and dispersive components of surface energy were determined according to the Owens–Wendt model [47].

Uniaxial tensile tests were performed on IL-modified PMMA samples in order to determine the Young’s modulus and the strain at break. The dumbbell-shaped specimens were tested using an MTS 2/M eletromechanical testing system (MTS, Eden Prairie, MN, USA) at 295 K under 50% relative humidity (RH) with a crosshead speed of 2 mm·min^−1^. In addition, in order to analyze more deeply the crazing mechanism, the samples were strained under a uniaxial tensile stress at different levels of strain (i.e., ranging from 20% to 80%) to be observed by optical microscopy (OM).

### 2.2. Processing of IL-Modified PMMA

PMMA was combined with 2 wt.% of ionic liquid ((C_4_mIm)(PF_6_), (N_1,1,1,4_)(PF_6_), (N1,1,1,6)(Br), or (N1,1,1,6)(TFSI)) in molten state using a 15 g capacity microextruder (DSM 15) with co-rotating twin screws. PMMA/IL mixtures were processed with the following parameters: 5 min at 100 rpm at a temperature of 180 °C and injected at 90 °C in order to generate samples for mechanical tests.

## 3. Results & Discussion

### 3.1. PMMA/IL Interactions

**Figure 2:**
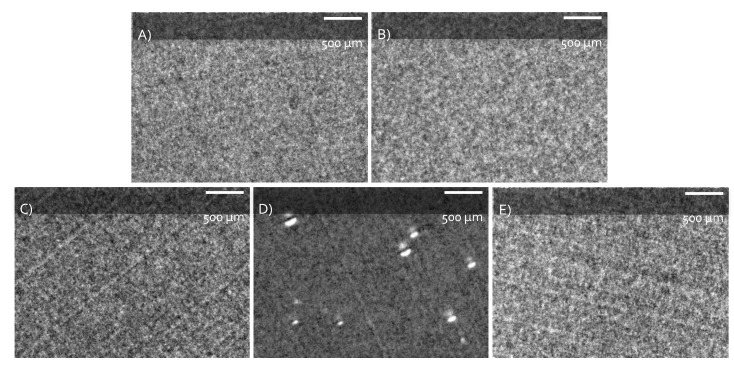
TEM micrographs of neat PMMA and IL-modified PMMA materials: (**A**) neat PMMA; (**B**) PMMA/(C_4_mIm)(PF_6_); (**C**) PMMA/(N_1,1,1,4_)(PF_6_); (**D**) PMMA/(N_1,1,1,6_)(Br); and (**E**) PMMA/(N_1,1,1,6_)(TFSI) (2 wt.% IL).

The miscibility of ILs in the PMMA matrix after melt mixing was examined by TEM. The TEM micrographs of neat PMMA and IL-modified PMMA materials are shown in Figure 2. The first conclusion is that the evidenced morphologies were in the nanometer range, which can explain why the IL-modified PMMA blends kept the initial PMMA optical properties.

TEM micrographs did not show large differences between PMMA, PMMA/(C_4_mIm)PF_6_), PMMA/(N_1,1,1,4_)PF_6_), and PMMA/(N_1,1,1,6_)(TFSI) morphologies. Such a phenomenon can be explained by the excellent miscibility of such imidazolium and ammonium ILs into the PMMA matrix [48]. On the other hand, one can notice on TEM micrographs of PMMA/(N_1,1,1,6_)(Br) the presence of tiny voids (with about 0.5 μm in diameter) scarcely dispersed in the PMMA matrix. This suggested a poor miscibility of the (N_1,1,1,6_)(Br) ionic liquid in PMMA.

**Figure 3:**
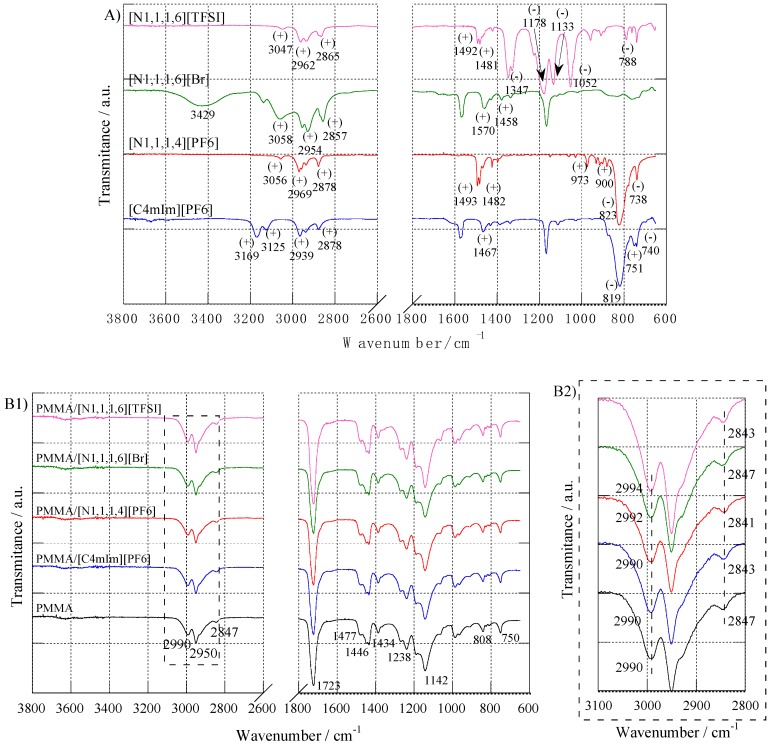
FTIR spectra of: (**A**) neat imidazolium and ammonium-based ILs, (+) and (−) captions highlight the main absorption bands for the cation and anion, respectively; (**B1**) neat PMMA and IL-modified PMMA materials; and (**B2**) displays the same spectra ranging only from 3100 to 2800 cm^−1^).

To provide a better understanding of the type of intermolecular interactions between PMMA and imidazolium or ammonium-based ILs, FTIR spectroscopy was used. FTIR spectra of ILs, PMMA, and the different IL-modified PMMA materials are given in Figure 3.

The ammonium and imidazolium ionic liquids were well characterized by the absorption bands at 2939 and 2878 cm^−1^, attributed to the C–H stretching vibration, and by the bands at 1467 and 751 cm^−1^, corresponding to the C=N and C–N stretching, respectively (Figure 3A). The symmetric as well as asymmetric stretching of the (PF_6_)^−^ anion appeared at 819 and 740 cm^−1^, respectively [41]. For (N_1,1,1,4_)(PF6), the absorption bands associated to the anion shifted to 823 and 738 cm^−1^. Despite the different lengths of the alkyl chains of ammonium cations (i.e., (N_1,1,1,4_)^+^ and (N_1,1,1,6_)^+^), the characteristic absorption bands were similar (Figure 3): C–H stretching was evidenced in the 3050–2860 cm^−1^ range; C–H bending at 1490–1480 cm^−1^; and at the lowest wavelengths, 970 and 900 cm^−1^, the characteristic vibrations of the C–N–C torsion bonds occurred. Concerning (N_1,1,1,6_)(Br) IL, the broad absorption band from 3600 to 3000 cm^−1^ was related to the water uptake, due the more pronounced hydrophilic character of this IL, which is associated to the (Br)^−^ anion. On the other hand, for the (N_1,1,1,6_)(TFSI) IL, the strong hydrophobicity of the (TFSI)^−^ anion prevents water uptake, and there was no evidence of residual water. The same conclusion could be made for (PF_6_)^−^ -based ILs [49]. The (TFSI)^−^ anion was characterized by strong absorption bands between 740 and 1350 cm^−1^: at 1347, 1328, and 1133 cm^−1^ for the SO_2_ group; from 1226 to 1178 cm^−1^ for the –CF_3_ group; and at 1052 and 788 cm^−1^ for S–N–S and C–S vibrations, respectively [50,51]. 

Figure 3B reports the spectra of PMMA and IL-modified PMMA materials. For PMMA, absorption bands appearing at 2990 and 2847 cm^−1^ corresponded to the asymmetric and symmetric stretches of C–H in the CH_3_ group, respectively. At 2950 cm^−1^, the –CH_2_– groups of the polymer backbone displayed their asymmetric stretching vibration mode. The C=O vibration modes were evidenced at 1723 (stretching), 808 (in-plane banding), and 750 cm^−1^ (out of-plane bending). The –CH_3_ deformation modes (1477, 1446, and 1434 cm^−1^), twisting (1142 cm^−1^), and the C–O bond stretching mode at 1238 cm^−1^ were also present.

For FTIR spectra of all IL-modified PMMA materials, one can notice that only the vibrational bands assigned to PMMA were observed according to the very small amount of IL added to PMMA. Nevertheless, it is possible for IL-modified materials to evidence shifts in the absorption bands related to methyl stretching vibrations (from 3100 to 2800 cm^−1^—see Figure 3B2). Thus, whatever the IL used, strong interactions between PMMA and IL took place.

In fact, according to the PMMA structure, the negative charge delocalized between the two oxygens of the methacrylate group induced a positive charge on the hydrogens of the methyl group. As a consequence, the (PF_6_)^−^, (Br)^−^, and (TFSI)^−^ anions can interact with the –OCH_3_ groups of PMMA (especially for (PF_6_)^−^ and (TFSI)^−^ because of the strong electronegativity of fluorine). As a consequence, one can consider that the compatibility between PMMA and the IL is dictated by the nature of the anion rather than the cation. In fact, this conclusion was indicated by Ueno et al. who analyzed the solubility of PMMA in several ILs. The authors demonstrated that the interactions between the polymer and ILs were governed by the anion structure, whereas the cation had secondary effects [52].

As reported in Figure 3B2 for PMMA/(N_1,1,1,6_)(TFSI), both C–H symmetric and asymmetric stretching absorption bands shifted by 4 cm^−1^ compared to the ones of neat PMMA. On the other side, for PMMA combined with (C_4_mIm)(PF_6_) or (N_1,1,1,4_)(PF_6_), only a shift of the C–H symmetric vibrational band could be evidenced (i.e., from 2847 cm^−1^ for neat PMMA to 2843 cm^−1^ for PMMA/(C_4_mIm)(PF_6_), and 2841 cm^−1^ for PMMA/(N_1,1,1,4_)(PF_6_)). No significant difference could be noticed between FTIR spectra of neat PMMA and PMMA/(N_1,1,1,6_)(Br). From these observations, one can conclude that (TFSI)^−^ displayed higher interactions with PMMA, whereas no specific interactions were created between (N_1,1,1,6_)(Br) and PMMA. These conclusions are in agreement with the literature, which reports that PMMA and ILs interact via van der Waals forces, and that (TFSI)^−^ exhibits the highest affinity with PMMA among a series of anions according to its high hydrophobicity [52,53,54].

Differential scanning calorimetry was also carried out to analyze the consequences of these intermolecular interactions between PMMA and ionic liquids on the Tg of PMMA (Table 2).

The Tg of PMMA remains the same after the addition of (N_1,1,1,6_)(TFSI) ionic liquid, whereas a decrease was noticed for the other IL-modified PMMA materials. As expected from the conclusions on the poor interactions of (N_1,1,1,6_)(TFSI) with PMMA, no plasticization occurred [24]. In the opposite, the incorporation of ILs such as (N_1,1,1,4_)(PF6) and (N_1,1,1,6_)(Br)^−^ into PMMA led to a slight decrease of the Tg (4 °C). Comparing the cation structures, it is evident that the acid hydrogen of the imidazolium ring (N = CH − N) can interact with the carboxyl group, enhancing the compatibility between the IL and polymer matrix. On the other hand, the Tg for PMMA/(C_4_mIm)(PF_6_) decreased about 7 °C compared to PMMA because of the specific interactions between PMMA and both (C_4_mIm)^+^ and (PF_6_)^−^, which compete with the intermolecular interactions between the polymer chains. As previously mentioned, many works described the use of ILs as plasticizing agents of polymer materials, especially because of their similar characteristics to conventional plasticizers, such as low volatility, low leachability, high temperature stability, and compatibility with polymers [43,55]. Moreover, ideal plasticizers possess a solvating group located internally rather than as terminal groups. Thereby, the charged structures of ILs are not much different [37].

**Figure 4:**
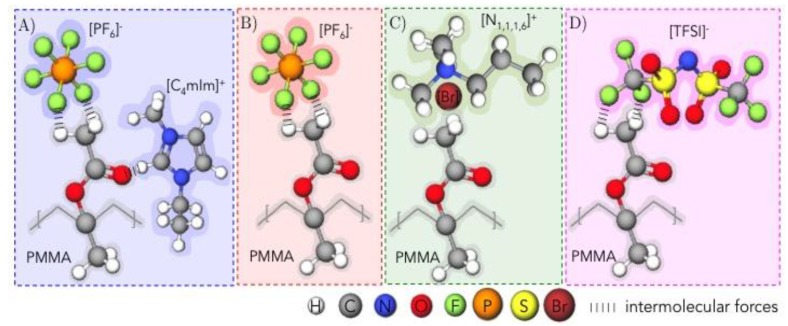
Schematic representations of the interactions between the methyl group of PMMA (–OCH_3_) and (**A**) (C_4_mIm)(PF_6_), (**B**) (N_1,1,1,4_)(PF_6_), (**C**) (N_1,1,1,6_)(Br), and (**D**) (N_1,1,1,6_)(TFSI) ILs.

Based on FTIR and DSC analyses, the respective interactions between PMMA and ILs can be summarized as reported in Figure 4. (As no interactions between PMMA and ammonium cations occur, the structures of (N_1,1,1,4_)^+^ and (N_1,1,1,6_)^+^ for (N_1,1,1,4_)(PF_6_) and (N_1,1,1,6_)(TFSI) ionic liquids are not represented.) On the other hand, (N_1,1,1,6_)(Br) is represented as a ion pair to highlight its poor interactions with PMMA. 

### 3.2. Consequences of the Presence of ILs on the Thermal Stability of PMMA

**Figure 5:**
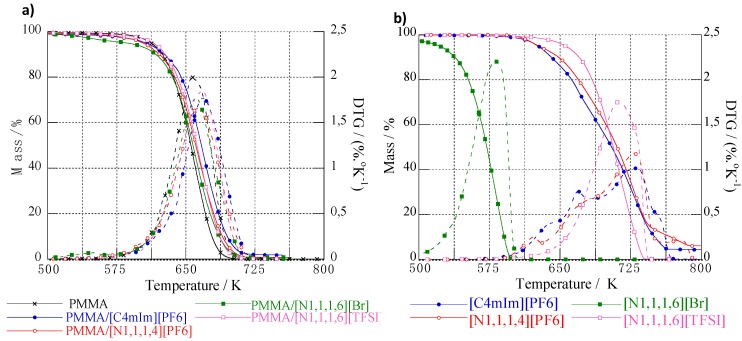
TGA (**a**) and DTG (first derivative) (**b**) traces of imidazolium- and ammonium-based ILs and IL-modified PMMA materials.

The thermal stability, determined by TGA, of neat ILs, neat PMMA, and IL-modified PMMA materials is shown in Figure 5.

#### 3.2.1. Thermal Stability of ILs

Among ILs, (N_1,1,1,6_)(TFSI) displayed the better thermal stability (i.e., higher than (C_4_mIm)(PF_6_) and (N_1,1,1,4_)(PF_6_)). The thermal stabilities of the counter anions can be ranked in the following order: (TFSI)^−^ > (PF_6_)^−^ >> (Br)^−^, which is in agreement with the literature. In fact, it is well known that the thermal stability of ILs is primarily dependent on the hydrophobicity of the anion, which could be associated with its nucleophilicity [56,57]. Indeed, the thermal decomposition of ILs have been attributed to the decomposition of the cation induced by nucleophilic attack of the anion [58,59,60]. Thus, the poor nucleophilicity of (TFSI)^−^ and (PF_6_)^−^ confers a high thermal stability to the IL [61].

In addition, other works highlighted that the thermal degradation of ILs is mainly controlled by the chemical nature of the counter anion [62,63]. Our results show a decrease of 13 K of the T_onset_ of (N_1,1,1,4_)(PF_6_) compared to (C_4_mIm)(PF_6_), which demonstrates clearly the effect of the nature of the cation. Usually, imidazolium cations are considered more thermally stable than the corresponding ammonium ones. Such an effect can be related to the fact that the thermal treatment of imidazolium cations induces rearrangements in the 1-substitute imidazole groups to prevent ring scission, whereas ammonium cations undergo Hofmann’s elimination under heating [61,64,65].

It is also important to notice that several works reported, as a general feature, that the longer the alkyl chain length, the less thermally stable the IL, as a longer alkyl chain confers an increased stability to the carbocation [56]. However, our results do not show any evidence about the effect of the alkyl chain of the cation on the thermal properties of ILs. (N_1,1,1,6_)(TFSI), having the longer alkyl chain, is the most stable compound. Such evidence contradicts the expectation that the imidazolium-based IL ((C_4_mIm)(PF_6_)) is more thermally stable than the ammonium ones. Overall, a clear effect of the nature of the anion on the thermal stability is observed for such ILs.

#### 3.2.2. Thermal Stability of IL-Modified PMMA Materials

The lowest values for T_max_, T_onset_, and T_final_ were related to neat PMMA, and improved thermal stability was achieved for IL-modified PMMA materials (Table 3). PMMA/(C_4_mIm)(PF_6_) displayed the highest thermal stability compared to PMMA/(N_1,1,1,4_)(PF_6_) and PMMA/(N_1,1,1,6_)(TFSI). The main information obtained from TGA analyses for neat PMMA and IL-modified PMMA materials was the good compatibility between PMMA and (C_4_mIm)(PF_6_), (N_1,1,1,4_)(PF_6_), and (N_1,1,1,6_)(TFSI), as a single-step of degradation was evidenced. In the opposite, the use of (N_1,1,1,6_)(Br) as a modifying agent for PMMA led to the appearance of an additional degradation peak at lower temperatures (about 553 K). In fact, this first step occurred in the same temperature range for the thermal decomposition of neat (N_1,1,1,6_)(Br). Such a phenomenon evidences the nonmiscibility of this IL with PMMA and confirms the conclusions issued from TEM analyses.

### 3.3. Surface Properties of IL-Modified PMMA

The influence of the introduction of ILs on the surface properties of IL-modified PMMA materials was studied by a sessile drop method. The data values obtained for neat PMMA and IL-modified PMMA materials are summarized in Table 3.

Whatever the chemical nature of ILs, their incorporation into the PMMA matrix induced an increase in the total surface energy. The major contribution to this phenomenon was the significant increase in the dispersive component combined with a decrease in the nondispersive component for IL-modified PMMA materials. This increase in the total surface energy lies on the nature of interactions acting at the surface [66]. In fact, in the case of PMMA, only van der Waals forces were involved, whereas Coulomb forces were superimposed for IL-modified PMMA surfaces. The changes observed with the nature of the IL can be explained by their chemical structures. In fact, ILs are usually considered as moderate polar compounds due to their 3D structuration (i.e., a polar core surrounded by dispersive domains). Thus, an increase in the alkyl chain length of the cation and in the anion size leads to a decrease in the surface energy of the IL, especially because of the ion charge [67]. As shown by Santos et al., for various imidazolium-based ILs, the dispersive domain increases when the alkyl chain increases. Thus, the number of van der Waals interactions between ion pairs increases, while the Coulombic forces remain constant [68]. Thus, as (N_1,1,1,6_)^+^ has a longer alkyl chain than the butyl chains present in (C_4_mIm)^+^ and (N_1,1,1,4_)^+^ cations, a decrease in the ratio of Coulomb to van der Waals forces is expected (i.e., a slighter decrease in the surface energy of materials prepared with (N_1,1,1,6_)^+^-based ILs is induced). In addition, we can assume that the lowest surface energy obtained for PMMA/(N_1,1,1,6_)(TFSI) is due to the additional effect in this balance of intermolecular forces of the large [TFSI]^−^ anion compared to the small (Br)^−^ anion. The nondispersive components of all IL-modified PMMA materials were lower than the one obtained for neat PMMA, as the incorporation of ILs led to more hydrophobic materials. However, it is important to notice that the nondispersive components for PMMA/(C_4_mIm)(PF_6_), PMMA/(N_1,1,1,4_)(PF_6_), and PMMA/(N_1,1,1,6_)(TFSI) were quite similar compared to the higher value obtained for PMMA/(N_1,1,1,6_)(Br). These results are in agreement with the expected phenomena, as it is widely reported in the literature that [PF_6_]^−^ and (TFSI)^−^ could be considered as hydrophobic anions, while (Br)^−^ is considered hydrophilic [69]. In fact, the anion could be used to tailor the hydrophobicity/hydrophilic balance of the IL, but the size of the cation could also play a role [38,39].

### 3.4. Mechanical Properties of IL-Modified PMMA

**Figure 6:**
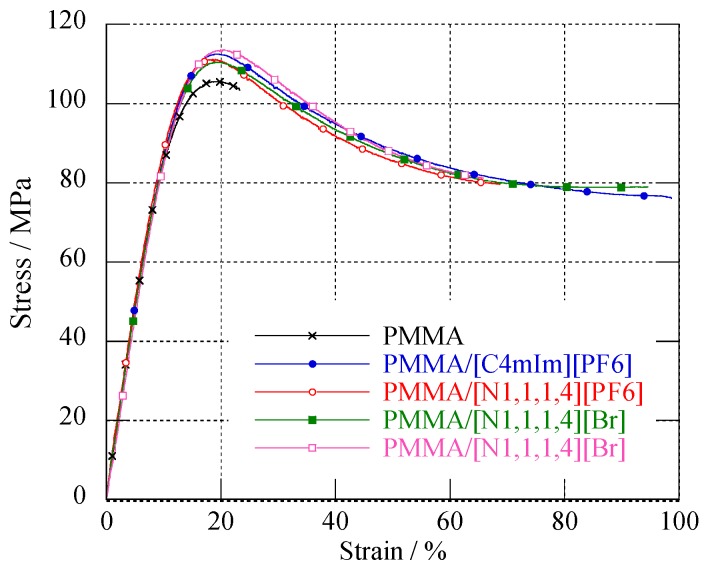
Strain–stress curves in uniaxial tension for neat PMMA and IL-modified PMMA materials (295 K, 50% RH, 2 mm·min^−1^).

Figure 6 reports the mechanical curves of neat PMMA and IL-modified PMMA materials (see Table 4 summarizing the Young’s modulus as well as elongation at break for the various materials).

PMMA is considered as a rigid and brittle polymer having a high Young’s modulus and low elongation at break [1,70]. Thus, usually, plasticizers are added to the polymer in order to enhance the ability of the material to sustain large deformations and improve toughness [71]. As shown in Table 4, a slight plasticizing effect of the ILs could be observed, whereas a very large increase in the strain at break was obtained for IL-modified PMMA materials compared to neat PMMA, whatever the nature of the added ionic liquid was. In addition, one can notice that for all the IL/PMMA materials, the yield stress remained similar, whatever the ionic liquid nature was, and was slightly higher than the one of the neat PMMA.

In fact, the addition of 2 wt.% of IL did not significantly impact the Young’s modulus of IL-modified PMMA. These results are similar to those reported by Scott et al., who studied PMMA plasticized with two imidazolium-based ILs at higher concentrations [43,44]. In fact, these authors reported that the Young’s modulus of PMMA is the same with the addition of 10 wt.% of IL, whereas it decreases dramatically for IL contents from 20 to 50 wt.%. Nevertheless, the lowest value of Young’s modulus obtained for PMMA/(N_1,1,1,6_)(Br) could be attributed to the poor interactions between this IL and PMMA, as reported previously. This fact leads to some exudation of the IL and voids formation. 

As mentioned, the presence of ILs in PMMA strongly influences the strain at break of the material. In a similar work done for polyvinylchloride, Rahman et al. reported the ability of an ILs series to improve the flexibility of PVC [37]. In their work, the strain at break of the various IL-plasticized PVC was in the range from 2% to 94% depending on the chemical nature of IL (i.e., much higher than that of the strain at break obtained for neat PVC (1.4%)). In our work, we demonstrated very clearly that even larger improvements could be achieved with the addition of IL into PMMA without main changes to the Young’s modulus. Except for PMMA/(N_1,1,1,6_)(Br), the extent to which the different ILs imparted plastic deformation of PMMA clearly lies with the Tg values (shown Table 2). In fact, PMMA/(C_4_mIm)(PF_6_) material, which has the lowest Tg, displayed the highest strain at break, whereas PMMA/(N_1,1,1,4_)(PF_6_) and PMMA/(N_1,1,1,6_)(TFSI) materials, having similar Tgs, displayed a strain at break of about 280%.

According to these observations on Young’s modulus and yield stress, it is obvious that the presence of IL mainly influenced the plastic deformation mechanisms (i.e., crazing- and/or shear bands-based phenomena).

### 3.5. Influence of ILs on the Deformation Phenomena of IL-Modified PMMA

In order to have a better understanding of the role of the IL on the deformation processes of IL-modified PMMA materials, the initiation and growth crazing mechanisms have been investigated The process of deformation and fracture of polymers under mechanical stress involves three main steps: the first one occurs at the molecular scale involving polymer chain re-arrangements, conformations changes, disentanglements, and scissions; the second step is governed by the appearance of crazes (crazing initiation, growth, and breakdown) at the microscale level, followed by initiation and propagation of microcracks; and the final step occurs at the macroscopic level with the material failure induced by the extension of cracks [72]. 

**Figure 7:**
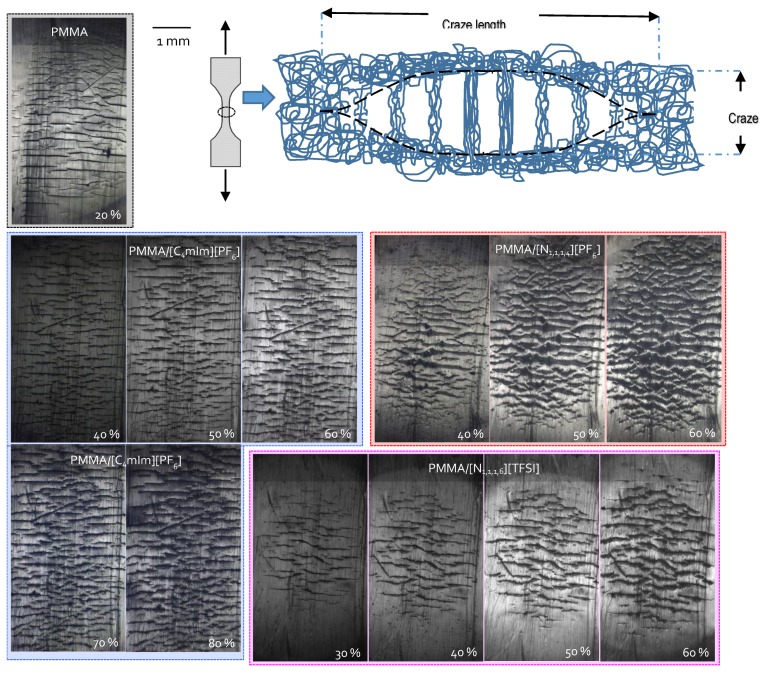
OM micrographs of neat PMMA and IL-modified PMMA materials at different tensile strain levels.

Under uniaxial tension, crazes propagate perpendicular to the direction of uniaxial stress and grow by extension and break of internal fibrils under specific conditions (see the scheme of a craze in Figure 7) [72,73]. Thus, the crazing mechanism of IL-modified PMMA materials was followed as a function of strain under uniaxial tensile stress. Figure 7 reports the optical microscopy micrographs of neat PMMA and IL-modified PMMA materials at different strain levels. In order to provide a quantitative analysis of the crazing process, the values for the average craze length and width were recorded as a function of the tensile strain (from 20% to 80% strain) (Table 5). Neat PMMA has a strain at break that is too low to sustain large tensile strains as the IL/PMMA materials. Thus, the crazes characteristics are reported only for 20% strain. 

As observed on OM micrographs, in the case of the neat PMMA, the appearance of crazes preceded the fracture to an extremely limited extent, whereas for IL-modified PMMA materials, the crazing growth mechanism was considerably slower and depended on the chemical nature of the IL used to modify PMMA.

It is known that the appearance and growth of crazes is an efficient phenomenon for toughening thermoplastics. In fact, craze growth plays a key role by absorbing energy from surface creation and chain pull out during the deformation process [74,75,76,77]. Thus, the generation of numerous nucleation sites of crazing enhances the amount of energy absorbed and contributes efficiently to the material toughness by delaying fracture. It is also well known that by enhancing the chain mobility, usually by increasing temperature, crazing initiation and the extension of the internal fibrils from chain pull out are facilitated [74,75]. It was previously demonstrated that ILs lead to a decrease in the intermolecular interactions between polymer chains. In fact, the presence of ILs delays the strain at which the first crazes appear. Observable crazes appeared at 20% of strain for neat PMMA, whereas 30% of strain must be reached for (N_1,1,1,6_)(Br)- and (N_1,1,1,6_)(TFSI)-modified PMMA and 40% for (C_4_mIm)(PF_6_)- and (N_1,1,1,4_)(PF_6_)-modified PMMA. 

On the other hand, comparing the craze widths and lengths as a function of strain could give information on the effect of the presence of ILs on the toughening mechanisms of IL-modified PMMA. The presence of (C_4_mIm)(PF_6_) IL clearly induced the appearance of the smallest crazes in PMMA for a given strain but also led to the slowest crazes growth mechanism. One can mention that for 60% of strain, the values for the average craze width and length of the PMMA/(C_4_mIm)(PF_6_) blend are smaller than the those of the neat PMMA at 20% of strain. These craze parameters (craze widths and lengths) displayed intermediate values for PMMA/(N_1,1,1,4_)(PF_6_) and (N_1,1,1,6_)(TFSI) blends but considerably large ones for the PMMA/(N_1,1,1,6_)(Br) blend. In addition, the special role of the (C_4_mIm)(PF_6_) ionic liquid could be evidenced from the craze density (i.e., crazes per mm^−2^), compared to the neat PMMA and the other PMMA/IL blends. This peculiar behavior can be attributed not only to the more efficient plasticizing effect of (C_4_mIm)(PF_6_) but also to its better miscibility (see Table 3). Compared to the effect of (C_4_mIm)(PF_6_), which induces a high strain at break associated to the plasticizing effect of the imidazolium-based IL, the introduction of (N_1,1,1,6_)(Br) did not induce any plasticization of PMMA/(N_1,1,1,6_)(Br) according to its poorest miscibility (Table 2). Hence, the tiny voids might explain the high strain at break observed for PMMA/(N_1,1,1,6_)(Br). As a consequence, the domains of (N_1,1,1,6_)(Br) observed by TEM in the PMMA are supposed to act as stress concentration zones inducing a failure at lower strain. In fact, up to 70% of strain, craze lengths and widths considerably increase, whereas the craze density decreases; this phenomenon is mainly governed by the propagation and coalescence of the previously formed crazes. 

As a conclusion, crazing initiation and propagation analyses clearly evidence the effect of the nature of the IL on the mechanical properties of IL-modified PMMA. For PMMA/(C_4_mIm)(PF_6_), PMMA/(N_1,1,1,4_)(PF_6_), and PMMA/(N_1,1,1,6_)(TFSI), a substantial increase in the strain at break was due to the plasticizing effect induced by the addition of IL, whereas for PMMA/(N_1,1,1,6_)(Br), the poor miscibility leading to low cohesion (N_1,1,1,6_)(Br) IL inclusions acted as stress concentration zones, which limited the strain break value of the corresponding blend.

## 4. Conclusions

The effect of the introduction of a low amount (2 wt.%) of imidazolium- and ammonium-based ILs in PMMA was evaluated in order to highlight the role of the cation and the anion on the final properties of the blends. Morphology analyses suggest a good miscibility between (C_4_mIm)(PF_6_), (N_1,_1,1,4)(PF_6_), and (N_1,1,1,6_)(TFSI) ionic liquids with PMMA. On the other hand, a poor interaction between PMMA and (N_1,1,1,_6)(Br) IL was observed, which can be explained by the strong cohesion between the ions of this IL in preventing interactions with the polymer. FTIR spectroscopy evidences that specific van der Waals interactions exist between the highly miscible ILs (i.e., (C_4_mIm)(PF_6_), (N_1,1,1,4_)(PF_6_), and (N_1,1,1,6_)(TFSI)) and the PMMA chains (due to the anion of the IL and the methyl groups of PMMA). In addition, the extent of these interactions contributes to the plasticizing effect of the ILs, as demonstrated by the slight decrease of the glass transition temperature. As no change on the Tg of PMMA/(N_1,1,1,6_)(Br) was noticed compared to neat PMMA, blends based on (N_1,1,1,4_)(PF_6_), (N_1,1,1,6_)(TFSI), and(C_4_mIm)(PF_6_) display an increase in the polymer chain mobility, as evidenced by a decrease of Tg. One can conclude that the specific interactions between PMMA and both [C_4_mIm]^+^ and [PF_6_]^−^ ions can explain the larger plasticizing effect induced by (C_4_mIm)(PF_6_) IL on PMMA.

The stronger the intermolecular interaction between IL and PMMA, the more thermally stable the PMMA/IL blends are, as evidenced by thermal analysis. These conclusions are issued from the increase of the activation energies, E_a_, of the thermal degradation, which suggests that the ILs can stabilize the radicals released during thermal degradation of PMMA. ILs positively influence the mechanical properties of the IL-modified PMMA blends. In fact, very limited decreases of the Young’s modulus combined with improved strain at break were observed and could be related to the plasticizing effect imparted by imidazolium and ammonium-based ILs on PMMA, except for PMMA/(N_1,1,1,6_)(Br). In addition, crazing initiation and propagation investigations clarified the influence of the IL on the mechanical behavior of PMMA. For PMMA/(C_4_mIm)(PF_6_), PMMA/(N_1,1,1,4_)(PF_6_), and PMMA/(N_1,1,1,6_)(TFSI), the large increase of the strain at break of the IL-modified PMMA could be attributed to the plasticization effect of the IL. In the opposite direction, for PMMA/(N_1,1,1,6_)(Br), which was evidenced by TEM to be nonmiscible in PMMA, the intermediate behavior could be attributed to the heterogeneity of the IL-modified PMMA blend, as (N_1,1,1,6_)(Br)-rich domains act as stress concentration zones (i.e., defects). As a conclusion, imidazolium- and ammonium-based IL can be proposed as efficient additives of PMMA, acting as processing aids in melt extrusion, and they lead to PMMA-like materials having improved thermal and mechanical behaviors. 

## Figures and Tables

**Table 1:** Designation of imidazolium and ammonium ILs used in this work.

Designation	Cation	Anion	T_m_ (K)	M_m_ (g·mol^−1^)
(C_4_mIm)(PF_6_)	1-butyl-3-methylimidazolium	Hexafluorophosphate	283	284
(N_1,1,1,4_)(PF_6_)	N-trimethyl-N-butylammoniun	Hexafluorophosphate	393	289
(N_1,1,1,6_)(Br)	N-trimethyl-N-hexylammonium	Bromide	268	224
(N_1,1,1,6_)(TFSI)	N-trimethyl-N-hexylammonium	Bis(trifluoromethanesulfonyl)imide	308	424

**Table 2:** Effect of imidazolium- or ammonium-based ILs on the Tg (DSC) and thermal stability—T_onset_, T_max_, and T_final_ corresponding to the starting, maximum, and end of degradation—considering the first derivative of the weight loss vs. temperature, respectively.

Material	T_g_/K	T_onset_/K	T_max_/K	T_final_/K
PMMA	367	614	656	691
PMMA/(C_4_mIm)(PF_6_)	360	627	667	708
PMMA/(N_1,1,1,4_)(PF_6_)	364	617	662	705
PMMA/(N_1,1,1,6_)(Br)	363	502 ^a^/614 ^b^	548 ^a^/659 ^b^	693
PMMA/(N_1,1,1,6_)(TFSI)	367	619	666	699

^a,b^ correspond to values obtained for the first and second step of degradation, respectively.

**Table 3:** Determination of nondispersive and dispersive components of the surface energy of neat PMMA and IL-modified PMMA materials from contact angles with water and diiodomethane.

Sample	θ_H2O_ (°)	θ_CH2I2_ (°)	γ_t_ (mN·m^−1^)	γ^d^ (mN·m^−1^)	γ^nd^ (mN·m^−1^)
PMMA	71 ± 1	40.1 ± 1.7	40.7	30.3	10.4
PMMA/(C_4_mIm)(PF_6_)	72 ± 1	27.0 ± 1.1	45.3	40.1	5.2
PMMA/(N_1,1,1,4_)(PF_6_)	72 ± 1	26.6 ± 1.7	45.3	40.5	4.8
PMMA/(N_1,1,1,6_)(Br)	66 ± 1	34.0 ± 0.9	44.6	35.1	9.5
PMMA/(N_1,1,1,6_)(TFSI)	73 ± 1	40 ± 0.7	43.0	37.8	5.2

**Table 4:** Effect of ILs on the tensile properties of IL-modified PMMA at 295 K. (50% RH, 2 mm·min^−1^).

Material	Young’s modulus (MPa)	Strain at Break (%)
PMMA	953 ± 7	24 ± 1
PMMA/(C_4_mIm)(PF_6_)	939 ± 2	96 ± 3
PMMA/(N_1,1,1,4_)(PF_6_)	943 ± 5	70 ± 4
PMMA/(N_1,1,1,6_)(Br)	920 ± 7	92 ± 3
PMMA/(N_1,1,1,6_)(TFSI)	934 ± 2	68 ± 4

**Table 5:** Craze width, length, and density obtained for neat PMMA and IL-modified for different given strains during a uniaxial tensile test performed at 20 °C.

	Strain (%)	Craze Width (μm)	Craze Length (μm)	Craze Density (crazes·mm^−2^)
PMMA	20	256	415	770
PMMA/ (C_4_mIm)(PF_6_)	40	18	332	1060
	50	23	361	1030
	60	24	395	970
PMMA/ (N_1,1,1,4_)(PF_6_)	40	22	416	490
	50	30	499	520
	60	39	531	550
PMMA/ (N_1,1,1,6_)(TFSI)	40	24	285	340
	50	31	464	340
	60	39	535	350
